# Establishing the human functioning sciences sections in frontiers in rehabilitation sciences

**DOI:** 10.3389/fresc.2026.1768887

**Published:** 2026-04-09

**Authors:** Jerome Bickenbach, Dorothy Boggs, Birgit Prodinger, Gerold Stucki

**Affiliations:** 1 Schweizer Paraplegiker-Forschung, Nottwil, Switzerland; 2 Health and Disability Researcher, Consultant, London, United Kingdom; 3 Chair of Inclusive Health Care, Universitat Augsburg, Augsburg, Germany; 4 Faculty of Health Sciences and Medicine, Universitat Luzern, Lucerne, Switzerland; 5 Center for Rehabilitation in Global Health Systems, WHO Collaborating Center, University Lucerne, Lucerne, Switzerland

**Keywords:** functioning, health status indicator, human functioning sciences, ICF (international classification of functioning disability and health), rehabilitation

## Introduction

Functioning, a concept developed by the World Health Organization in its *International Classification of Functioning, Disability and Health*, (ICF) (1) describes the full lived experience of health, comprised by both biological health and the performance of activities of daily life, or lived health. As functioning describes a person's actual experience of their health, the notion can only be understood in light of their full life context or environment, from immediate physical surroundings, other people and their attitudes, wider environments and ultimately social institutions and resources. WHO has made it clear that, although health states are purely biological phenomena, what matters to people about their health is not only these biological processes but also the concrete impact on their daily lives: what they can do and be, the actions they perform and the life goals and aspirations they can achieve. As a scientific notion with practical applications, functioning requires both an underlying conceptual description or model, and a classification for operationalization and measurement. The ICF provides both.

Epidemiologically, functioning can be understood as the third indicator of health, augmenting the traditional indicators of mortality and morbidity. As an indicator of the lived experience of health, functioning identifies an essential component of our overall understanding of human health, both as an individual experience and as a population phenomenon; mortality and morbidity inform us of how health can be compromised, and functioning informs us what we actually do and can achieve in our lives in light of a health condition—that is, the lived experience of health.

As a classification, the ICF has created an international reference language for the collection of information about the lived experience of health. The ICF therefore complements and supplements the WHO's *International Classification of Diseases* (ICD) (2) and the *International Classification of Health Intervention* (ICHI) (3). WHO has developed and maintains these international standards in order to ensure comparability of international health information.

At the *micro* level, functioning information provides the common language for health professionals in their interactions with persons living with health conditions. At the *meso* level of service provision, functioning information monitors individuals' functioning levels along the continuum of care and across the life course, and is used as the basis for payment systems. Finally, at the *macro*-level, functioning information serves as a metric for population health complementing data on causes of death and morbidity. Such information is essential for informing policies and laws, as well as health and social services programming. Ultimately, these *micro*, *meso* and *macro* level applications provide data towards the development of a full epidemiology of functioning.

## The functioning revolution

The recognition that human functioning is our best operationalization of health aligns with what we now can predict to be future health trends. Population ageing and the increasing prevalence of non-communicable diseases, coupled with medical and technical advances in prevention and early diagnosis, as well as rising survival rates, will lead to a future in which people will live longer, but with more functioning difficulties. A focus on functioning therefore represents a much-needed paradigm shift at the wider health systems level. By incorporating functioning into health and social policies and related practices, we can move beyond preventing and treating diseases and focus more holistically on the full lived experience of health. This approach will enhance individuals' real-world abilities and elucidate the conceptual and empirical link between health, individual well-being and societal welfare (4).

In short, WHO's concept of human functioning constitutes a fundamental rethinking of health; it represents a revolution with wide-ranging consequences across all components and structures of both health and social systems (5). Indeed, this rethinking argues for a broader understanding of the health sector that merges and synthesizes existing health and social sectors. The functioning revolution will reveal a wide and diverse range of scientific and social opportunities, as well as simultaneously creating implementation challenges. However, in order to realize this functioning revolution there is a need to develop the science that investigates functioning itself—the field of Human Functioning Sciences.

## The human functioning sciences

**Figure 1:**
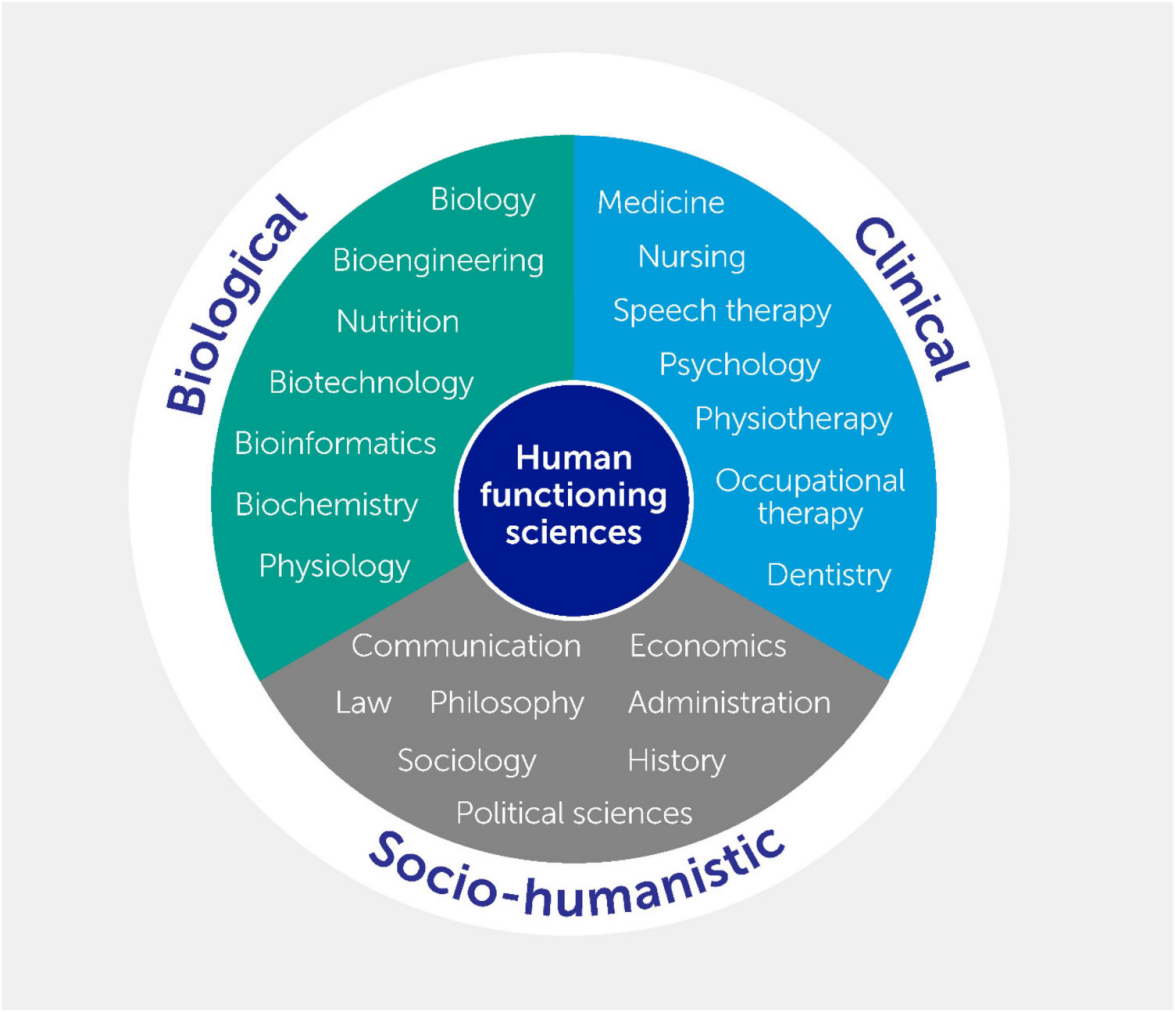
The human functioning sciences (5).

Given the conceptual breadth of functioning, the scientific field of Human Functioning Sciences relies on the broadest range of scientific disciplines representing the biomedical and technological, the clinical and socio-humanistic perspectives (6). Human Functioning Sciences is therefore a comprehensive and interdisciplinary scientific field (see Figure 1). This scientific field therefore encompasses what is usually thought to be the domains of both the health and social systems.

Within health systems, Human Functioning Sciences are of specific relevance to rehabilitation, as the primary aim of rehabilitation is to optimize functioning for people living with a health condition (7). But functioning as health indicator is also relevant to the other health strategies of prevention, promotion, cure and palliation (8). The scope of Human Functioning Sciences therefore comprises all health strategies relevant for individual and population health, as well as clinical and public health programs and practices, such as assistive technology.

Across the wider social system, functioning is also at the conceptual core of administrative procedures for disability assessment for benefits, for work capacity assessment and eligibility for social resources to ensure independent living and community integration.

More broadly still, functioning is a social indicator for determining the effectiveness of the societal response to functioning-based needs that are provided across a range of societal institutions, from labor, education, housing, communications, transportation, and city planning. Functioning can be used to describe and understand more profound change, from the impact of war, violence, poverty and environmental change on person's lived experience of health, beyond the risks of morbidity and associated mortality.

The functioning needs of people living with health conditions provide the rationale for and focus of the health-accommodating modifications in the built environment and so is foundational for universal design and accessible engineering and architecture. At the most general societal level, functioning is potentially the most relevant concept for understanding how to achieve equality of opportunities, human rights and ultimately overall well-being of persons across the life course (4).

## Current state of research in the human functioning sciences

Since the launch by WHO of the ICF in 2001, there has been an exponential growth of practical applications and scientific studies exploiting the functioning revolution in health care, health services, and the variety of wider societal institutions and structures described above. Methodological developments include: standards on what to document for specific health conditions, and generically, how to report functioning information on an interval scaled metric similar to the range of existing assessment tools and ICF- based assessment tools. These assessment tools include expert-assessed, person-reported, and electronically-extracted information, such as from sensors. The ICF serves as reference for the reporting of outcomes in intervention studies in the Cochrane collaboration and has served as reference for the description of targets for rehabilitation in the package of rehabilitation interventions developed by WHO (9), including outcomes for ageing population (10). Functioning has also been used as a starting point for epidemiological studies, worldwide with WHO Model Disability Survey (11), and more specifically into the lived experience exemplified by the Swiss Spinal Cord Injury Cohort Study (SwiSCI) and the International Spinal Cord Survey (InSCI) (12, 13).

In light of all these diverse developments, and in anticipation of the rapid expansion and broadening of these developments in the future, we believe the time is right for the conceptual development of and advocacy for Human Functioning Sciences. Providing a restrictive definition of Human Functioning Sciences at this point might limit the potential range, scope and innovation of this new scientific field. Instead, we and the editors of Frontiers believe that by encouraging the publication of articles that represent the development functioning-based research is the better approach. This approach facilitates the integration of existing developments from rehabilitation, assistive technology and other areas of health sciences, while also expanding the scope to other fields and areas where societal responses are tailored to the needs of people with diverse health problems.

This effort is being co-sponsored by the University of Lucerne, which has launched the Lucerne Initiative for Functioning Health and Well-being (LIFE), an initiative that aims at optimizing research into functioning, health, and well-being against the backdrop of increasing prevalence of chronic diseases, injuries, and population aging. The mission at LIFE is to promote a new understanding of health as functioning within society and to implement this understanding in healthcare systems, research, education, and practice. The activities of LIFE will include a yearly forum, a school for Human Functioning Sciences and LIFE-StARS, an international program for the standardized assessment and reporting of functioning using the ICF as a reference system. The LIFE community includes a worldwide faculty of professors and fellows, as well as practitioners with an interest in human functioning sciences (https://www.unilu.ch/en/life).

## Frontiers of rehabilitation sciences is now launching two sections on human functioning sciences

Recognizing the need to foster and provide a space to publish research to establish Human Functioning Sciences, *Frontiers in Rehabilitation Sciences* is now expanding the current Human Functioning Sciences into two distinct sections. The goal of these sections is to stimulate and strengthen research into the lived experience of people living with health conditions, as well as the development of the interdisciplinary scientific field of Human Functioning Sciences.

Over the next months and years, calls for contributions to Research Topics for the two sections will be made. The Research Topics for each section will be launched by a discussion Paper by the Journal's Field and the Specialty Section editors.

## Human functioning sciences: concept, awareness and applications of functioning

Any scientific or cultural advancement that incorporates a new way of thinking, a radical change in perspective or a new understanding of or approach to an established notions such as health, rehabilitation or well-being initially requires careful thought and conceptual clarification. This might involve an exploration in history of idea or cultural trends, or a precise definition based on unambiguous, necessary and sufficient conditions for the application of the new concept. There is no doubt that the foundational thinking that went into the development of the ICF was anticipated in rehabilitation, assistive technology and disability literature for decades—so there is a historical story to be told. At the same time, there is a need for further clarification of the ICF concept of functioning and a clearer definition, especially one that can be operationalized for data collection (14). Further development in this area depends on scaling up awareness of functioning among key stakeholders: the public at large, professionals within health and social practices, as well as researchers across diverse disciplines.

Yet, conceptual clarity and awareness raising are just the beginning. Functioning must also be implemented and evaluated in practice. The perceived added value of functioning, and its viability in practice and research, depends on demonstrating both the feasibility and the significance of its applications in practice. These applications apply at every level, the *micro*-level of clinical practice, the *meso*-level of institutional management, planning and financing, and the *macro*-level of policy and programs. Applications of functioning involve both the health sector (e.g., functioning in health information systems), the social sector (e.g., functioning in disability determination for social benefits or work capacity) and beyond (e.g., national statistical offices, education sector).

Submissions are invited under this section that contribute to the conceptual development, definition and refinement of the notion of functioning, and to awareness raising and implementation strategies emphasizing added-value applications of functioning to health and social sectors.

### Human functioning sciences: classification, measurement and epidemiology of functioning

Making the case for functioning as the third health indicator to augment the traditional health indicators of mortality and morbidity raises basic scientific concerns of description, classification and measurement. Although the ICF itself is an international classification, a standard for data collection, functioning information must be recorded in a manner that ensures interoperability across all relevant data settings. At the same time, these data must be collected and recorded in a manner that makes it possible for reliable and meaningful monitoring of individual and population functioning over time. Additionally, once functioning information is available, it should be utilized for answering epidemiological questions by using methodologies that are fit for purpose, and to quantitatively describe and predict individual and population trends over time. This may demand innovations in statistical analyses, explorations into machine-learning technologies, and, clearly, the application of AI at all levels.

As an officially-endorsed international classification under WHO's auspices, the ICF serves as international reference language. However, in order to fulfill requirements for interoperability, the ICF may require amendments or modifications to satisfy fundamental, ontological and classificatory standards. Much depends on the ontological rigor of the ICF classifications, not only for international up-take across national health systems and statistical offices, but also to ensure the descriptive accuracy and international validity of ICF classificatory language. These scientific classification and measurement standards must be meet for all applications of ICF as a classification. Arguably, considerable research still needs to be done in this area.

Submissions are invited under this section to contribute to the foundational preconditions of the measure of functioning—both at the individual and population levels—as well as to the conceptual and practical development of an epidemiology of functioning.
